# Predictive value of dental readiness and psychological dimensions for oral health-related quality of life in Croatian soldiers: a cross-sectional study

**DOI:** 10.3325/cmj.2012.53.461

**Published:** 2012-10

**Authors:** Stjepan Špalj, Davorka Perić, Magda Mlacović Zrinski, Martina Bulj, Darije Plančak

**Affiliations:** 1 Department of Dental Medicine, School of Medicine, University of Rijeka, Rijeka, Croatia; 2 Department of Military Medicine, Croatian Army, Zagreb, Croatia; 3 Department of Periodontology, School of Dental Medicine, University of Zagreb, Zagreb, Croatia

## Abstract

**Aim:**

To determine the predictive value of dental readiness and psychological dimensions for oral health-related quality of life (OHRQoL) in Croatian soldiers.

**Methods:**

The sample consisted of 402 consecutive soldiers aged 21 to 54 years classified into the following groups according to dental readiness: Class 1 – not requiring dental treatment (N = 54), Class 2 – unlikely to need emergency treatment within 12 months (N = 205), and Class 3 – very likely to need treatment within 12 months (N = 143). OHRQoL was assessed by the Oral Health Impact Profile and psychological dimensions by the Brief Symptom Inventory and Dental Anxiety Scale.

**Results:**

Multivariate analysis showed that Class 3 soldiers had higher frequency of psychological discomfort, psychological disability, and physical pain and handicap than Class 1 soldiers (*P* = 0.019). Multiple linear regression showed that longer military experience, higher level of dental anxiety, and dental unreadiness were significant predictors of lower OHRQoL (*P* < 0.050) but accounted for low variability. None of the single psychological symptomatic dimensions was a significant predictor of OHRQoL.

**Conclusion:**

Although this study found a moderate association between OHRQoL and clinical, military, demographic, and psychological variables, the significant predictors could be used as a basis for further research of clinical and psychosocial factors of OHRQoL.

General and oral health have multiple effects on the quality of life (QoL), which can be decreased by pain, discomfort, and difficulties in everyday physical activities, chewing, speech, hygiene, relaxation, and social contact (1-3). Oral health is an often neglected dimension of health, especially among soldiers, although it affects their QoL and combat readiness (4). An inadequately assessed combat readiness may reduce the effectiveness of the military unit and cause human risks and financial expenses due to transportation of soldiers to health facilities (5). Acute dental conditions may cause losses of over 18 000 man-days per division per year (6,7). Dental non-battle injury emergency rates averaged 16% during Vietnam War (8), and in US military units in peacekeeping missions in Bosnia and Herzegovina the rate of emergency dental conditions was 156-170 per 1000 soldiers per year (9).

Psychological personality dimensions might influence self-perceived QoL, thus potentially affecting the reported health impairment and combat disability. We hypothesized that the psychological dimensions that may affect the self-perception of OHRQoL were somatization, obsessive-compulsive symptoms, interpersonal sensitivity, depression, anxiety, hostility, phobic anxiety, paranoid ideas, psychoticism, and dental anxiety.

The studies of HRQoL in soldiers have so far been sporadic (10,11). There were studies that assessed the impact of oral health on combat readiness or the consequences of wartime events on oral health, but often overlooked the QoL assessment (6,12-14). To the best of our knowledge, no studies so far have assessed the effect of psychological dimensions on the self-perceived OHRQoL.

The aim of the research was to explore the predictive value of dental readiness and psychological dimensions for OHRQoL. The hypothesis was that OHRQoL was a specific dimension of combat readiness that was significantly correlated with clinically assessed dental readiness and psychological symptomatic dimensions.

## Material and methods

This cross-sectional study included 402 consecutive professional soldiers aged 21 to 54 years (mean age 35.3 ± 6.0), who during October and November 2010 attended the annual periodic health and fitness evaluation at the Croatian Army Medical Center. Nineteen participants were removed from the initial sample (n = 421) because of incomplete or improperly filled-out questionnaires (4.5%). Only male soldiers were included to obtain a homogenous sample, since there are very few women in the active military service.

The study was approved by the Croatian Ministry of Defense (No. 512M2-0301-10/3) and the Ethics Committee of the Zagreb University School of Dental Medicine (No. 05-PA-35-XVII-11.1.3.2/11), and each participant gave a written consent.

Sociodemographic data included age, educational level, years spent in the military service, and history of participation in international peacekeeping missions. The oral health assessment included intraoral dental examination, panoramic x-ray evaluation, and the risk-assessment of having a non-combat dental emergency according to Oral Health and Readiness Classification System (15). A total of 54 soldiers were classified as dental fitness Class 1 – not requiring dental treatment; 205 as Class 2 – unlikely to need emergency dental treatment within 12 months; and 143 as Class 3 – very likely to need emergency dental treatment within 12 months. Class 1 and 2 soldiers were considered as combat ready and worldwide deployable, and Class 3 as combat-unready (15).

OHRQoL was assessed by the self-administered questionnaire Oral Health Impact Profile (OHIP-14) and psychological dimensions by using the Brief Symptom Inventory (BSI) and Dental Anxiety Scale (DAS).

The Croatian version of Slade’s Oral Health Impact Profile (OHIP-14 CRO) showed good reliability in the psychometric validation in the general population (Internal consistency Cronbach α = 0.83, test-retest reliability intraclass correlation coefficient r = 0.94) (16). The questionnaire comprises 14 items grouped in 7 domains: functional limitation, physical pain, psychological discomfort, physical disability, psychological disability, social disability, and handicap. The Dental Anxiety Scale-Revised (DAS-R) comprises four questions with five offered answers with values from a = 1 to e = 5 and the value of the scale is obtained by summing up the values of the answers to all questions (17). The instrument was validated in the Croatian population and summed values of the scales from 4-8 indicate low dental anxiety, from 9-12 indicate moderate dental anxiety, and >13 indicate high dental anxiety (18).

The Brief Symptom Inventory (BSI) is an inventory of 53 self-report items that assess psychopathology and psychological distress (19). It provides multidimensional measures of psychological symptoms, measuring nine dimensions of symptoms: somatization, obsessive-compulsive, interpersonal sensitivity, depression, anxiety, hostility, phobia, paranoid ideas, and psychoticism. It enables the calculation of three global psychopathology indices, which indicate the overall psychological distress level – Global Severity Index (GSI), intensity of symptoms – Positive Symptom Distress Index (PSDI), and the number of self-reported symptoms – Positive Symptoms Total (PST) (19). The respondents rank the intensity of the experienced distress related to each of the 53 listed problems during the past week on a Likert-type scale from 0 = “not at all” to 4 = “very much” (20). BSI was developed for use in clinical and research conditions where a relatively short measure of the participant’s perceived symptomatology was needed (20). BSI has shown very good reliability in the American general population (internal consistency Cronbach α = 0.71-0.85 and test-retest reliability intraclass correlation coefficient r = 0.68-0.91) and in the Croatian general population (Cronbach α = 0.69-0.84) (21).

### Statistics

To determine the influence of demographic, military, and psychological variables on OHRQoL in participants grouped according to levels of dental readiness we used ANOVA, Bonferroni post-hoc test, and χ^2^ test. The Bonferroni was used since it is suggested that it has more power than other post-hoc tests when the number of comparisons is small (22). η^2^ and Cramer V were used as a measure of effect size. Discriminant analysis with discriminant canonical function was applied to assess which of the OHRQoL variables best discriminated the levels of dental readiness. Several models of multiple linear regressions were tested in order to determine whether demographic and military variables, dental fitness, and psychological symptomatic dimensions could serve as a predictor of OHRQoL. For the purpose of analysis, dental fitness was dichotomized as 0 = dentally ready (Class 1 and 2) and 1 = dentally unready (Class 3). A hierarchical approach was used in order to control the effects of theoretically assumed importance of predictors being added to the existing regression model. Predictors with hypothesized higher importance were entered first, those with no significant predictive value were removed, and new variables were added. This approach allowed us to control for the effects of variables of probably higher association when testing the variables that we were more interested in. Statistical analyses were performed using the SPSS 10.0 (SPSS Inc., Chicago, IL, USA) with the significance level set to *P* < 0.05.

## Results

Class 1 participants were significantly younger and had shorter military service than Class 2 and Class 3 participants (*P* < 0.050; Table 1). There was no significant difference in the level of education and previous participation in military missions (Table 1).

**Table 1:** Comparison of demographic and military variables, and self-perceived oral health between dental readiness classes

Variable	Dental readiness class			Total	*P*	Effect size
	1 (n = 54, 13.4%)	2 (n = 205, 51.0%)	3 (n = 143, 35.6%)	N = 402		
Age (mean ± standard deviation)	32.3 ± 6.1^a^	36.0 ± 5.9^b^	35.3 ± 5.8^b^	35.3 ± 6.0	<0.001*	0.041^‡^
Years in military service (mean ± standard deviation)	10.0 ± 6.0^a^	14.7 ± 5.8^b^	14.1 ± 6.3^b^	13.8 ± 6.2	<0.001*	0.061^‡^
Experience in international military missions (n, %)						
no	26 (48.1)	81 (39.5)	64 (44.8)	171 (42.5)		
yes	28 (51.9)	124 (60.5)	79 (55.2)	231 (57.5)	0.417†	0.066^§^
Educational level (n, %)						
primary	1 (1.9)	9 (4.4)	7 (4.9)	17 (4.2)		
secondary	37 (68.5)	156 (76.1)	111 (77.6)	304 (75.6)		
college/university	16 (29.6)	40 (19.5)	25 (17.5)	81 (20.1)	0.369†	0.073^§^
OHIP^¶^ sum score (mean ± standard deviation)	7.0 ± 6.2^a^	9.6 ± 6.9^b^	10.9 ± 6.6^b^	9.7 ± 6.8	0.001*	0.032^‡^
Global Severity Index (mean ± standard deviation)	0.2 ± 0.4	0.2 ± 0.4	0.3 ± 0.4	0.3 ± 0.4	0.614*	0.002^‡^
Positive Symptom Distress Index (mean ± standard deviation)	1.3 ± 0.4	1.2 ± 0.4	1.2 ± 0.3	1.2 ± 0.4	0.732*	0.002^‡^
Positive Symptoms Total (mean ± standard deviation)	9.0 ± 11.1	9.3 ± 12.4	10.9 ± 13.9	9.8 ± 12.8	0.476*	0.004^‡^
Dental Anxiety Scale (mean ± standard deviation)	7.1 ± 2.0^ab^	6.9 ± 2.6^a^	7.7 ± 2.8^b^	7.2 ± 2.6	0.032*	0.017^‡^

*ANOVA with Bonferroni post hoc test. Dental readiness groups that share the same superscript letters do not differ at *P* < 0.05. Significant values are in bold.

†χ^2^ test.

‡η^2^ squared.

§Cramer’s V.

¶OHIP – Oral Health Impact Profile-14 CRO.

**Figure 1:**
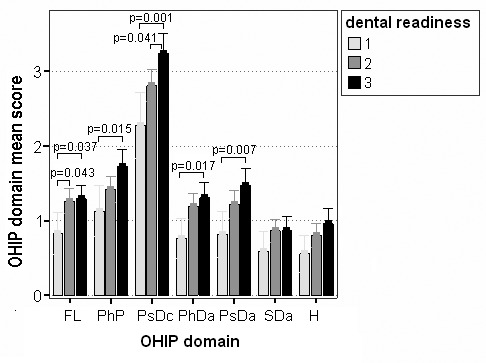
Differences in Oral Health Impact Profile domains between grades of dental readiness; ANOVA with Bonferroni post-hoc test; FL – functional limitation; PhP – physical pain; PsDc – psychological discomfort; PhDa – physical disability; PsDa – psychological disability; SDa – social disability; H – handicap.

OHIP sum score differed significantly between the participants with different levels of dental readiness. Soldiers with lower dental readiness had lower OHRQoL, however, the effect size was small, lower than 4% (*P* = 0.001, Table 1). Concerning OHIP domains, Class 1 participants had a lower frequency of functional limitations, physical pain, psychological discomfort, physical disability, and psychological disability than Class 3 participants (*P* < 0.050; Figure 1). Concerning the effect size, dental readiness level explained a small portion of variability in the OHIP domains. The effect size was in range from 1.7% for functional limitations to 3.7% for psychological discomfort, and 3.2% for the total OHIP score. Lower dental readiness was associated with lower OHRQoL, but this association was significant only for functional limitations, physical pain, and psychological discomfort (Table 2). Differences between dental readiness groups accounted for less than 16% of variation in the prevalence of discomfort/disability related to oral conditions (Table 2).

**Table 2:** Distribution of soldiers according to Oral Health Impact Profile (OHIP-14 CRO) subdomains and dental readiness

		No. (%) of soldiers with dental readiness class					
		1 (n = 54)	2 (n = 205)	3 (n = 143)	total	P*	Effect size^†^
Functional limitation	≤hardly ever	45 (83.3)	141 (68.8)	92 (64.3)	278 (69.2)		
	≥occasionally	9 (16.7)	64 (31.2)	51 (35.7)	124 (30.8)	**0.036**	**0.129**
Physical pain	≤hardly ever	41 (75.9)	152 (74.1)	87 (60.8)	280 (69.7)		
	≥occasionally	13 (24.1)	53 (25.9)	56 (39.2)	122 (30.3)	**0.016**	**0.143**
Psychological discomfort	≤hardly ever	47 (87.0)	166 (81.0)	99 (69.2)	312 (77.6)		
	≥occasionally	7 (13.0)	39 (19.0)	44 (30.8)	90 (22.4)	**0.007**	**0.157**
Physical disability	≤hardly ever	48 (88.9)	166 (81.0)	112 (78.3)	326 (81.1)		
	≥occasionally	6 (11.1)	39 (19.0)	31 (21.7)	76 (18.9)	0.239	0.084
Psychological disability	≤hardly ever	48 (88.9)	172 (83.9)	110 (76.9)	330 (82.1)		
	≥occasionally	6 (11.1)	33 (16.1)	33 (23.1)	72 (17.9)	0.093	0.109
Social disability	≤hardly ever	51 (94.4)	188 (91.7)	126 (88.1)	365 (90.8)		
	≥occasionally	3 (5.6)	17 (8.3)	17 (11.9)	37 (9.2)	0.317	0.076
Handicap	≤hardly ever	52 (96.3)	181 (88.3)	123 (86.0)	356 (88.6)		
	≥occasionally	2 (3.7)	24 (11.7)	20 (14.0)	46 (11.4)	0.128	0.101

*χ^2^ test. Significant values are in bold.

†Cramer's V.

There were no significant differences in global indices of psychopathology and intensity of psychological symptoms between levels of dental readiness, except for dental anxiety (Table 1). Class 2 participants had a significantly lower level of dental anxiety than Class 3 (*P* = 0.028; Table 1).

The discriminant analysis indicated that the levels of dental readiness were mostly discriminated by the following OHIP dimensions: psychological discomfort, psychological disability, physical pain, and handicap. These dimensions formed the first and only significant discriminant canonical function that accounted for 75.9% of variability (*P* = 0.019; Tables 3 and 4). In the multivariate discriminant space, Class 1 had significantly lower intensity of psychological discomfort, psychological disability, physical pain, and handicap than Class 3. A total of 39.8% of participants were correctly classified by discriminant function and the membership in the original class was retained by 59.3% of members of Class 1, 28.8% of members of Class 2, and 48.3% of Class 3. Assumptions for discriminant analysis (multivariate normality, homogeneity of covariance matrices, and linearity) were satisfied.

**Table 3:** Summary of canonical discriminant functions

Function	Eigenvalue	Percent of variance	Canonical correlation	Test of functions	Wilks' lambda	χ^2^	Degrees of freedom	*P*
1	0.053	75.9	0.224	1 through 2	0.934	27.024	14	**0.019**
2	0.017	24.1	0.129	2	0.983	6.607	6	0.359

**Table 4:** Standardized canonical discriminant function coefficients and pooled within-groups correlations between discriminating variables and standardized canonical discriminant functions

	Function	
Oral Health Impact Profile domains	1	2
Psychological discomfort	0.628 (0.848*)	-0.030 (0.211)
Psychological disability	0.436 (0.664*)	-0.268 (0.273)
Physical pain	0.407 (0.657*)	-0.727 (0.046)
Handicap	0.138 (0.468*)	-0.328 (0.202)
Functional limitation	-0.098 (0.431)	0.893 (0.683*)
Physical disability	0.061 (0.528)	0.587 (0.533*)
Social disability	-0.572 (0.252)	0.556 (0.462*)

*Significant correlations between discriminating variables and standardized canonical discriminant functions.

The first multiple regression model tested the significance of age, level of education, military service, participation in peacekeeping missions, and dental readiness in the prediction of OHRQoL based on OHIP sum score. The model was significant (*P* < 0.001), but accounted for a small part of variability (5.4%; Table 5). Military service and dental readiness were significant predictors of OHIP sum score (*P* < 0.050) and, with the control of other variables in the model, accounted for 2.1% and 1.3% of variability of OHIP, respectively. Lower OHRQoL was associated with longer military service and dental unreadiness.

**Table 5:** Multiple regression analysis models for prediction of oral health-related quality of life based on Oral Health Impact Profile sum score which used demographic and military variables and dental readiness as predictors

Predictor variable*	B^†^	Standard error	β^‡^	*P*	Correlation		
					zero-order	partial	semipartial
Constant	14.133	3.078		<0.001			
Age	-0.152	0.122	-0.134	0.213	0.131	-0.063	-0.061
Educational level	-1.144	0.728	-0.078	0.117	-0.089	-0.079	-0.076
Years in military service	0.351	0.118	0.319	**0.003**	0.192	0.148	0.145
Participation in international military missions (0 = no, 1 = yes)	-0.732	0.682	-0.053	0.284	-0.018	-0.054	-0.052
Dental readiness (0 = unready, 1 = ready)	-1.622	0.695	-0.114	**0.020**	-0.129	-0.116	-0.113

*R = 0.257; R2 = 0.066; Adjusted R2 = 0.054; F = 5.609; *P* < 0.001. Significant values are in bold.

†Unstandardized coefficient.

‡Standardized coefficient.

The correlation between OHRQoL and psychological dimensions was positive, significant, but weak in the span of 0.134 for psychoticism to 0.212 for obsessive-compulsive symptomatology (*P* < 0.050, zero-order correlations in Table 6). Although obsessive-compulsive symptoms showed the highest correlation with OHIP sum score in univariate analysis, dental anxiety was the only significant psychological predictor in multivariate analysis with the control of other predictors in the second model (Table 6). The model accounted for 12% of variability of OHIP; and military service, dental anxiety, and dental readiness were significant predictors that respectively accounted for 4%, 3%, and 1% of variability.

**Table 6:** Multiple regression analysis models for prediction of oral health-related quality of life based on Oral Health Impact Profile sum score which used psychological dimensions, years spent in military service and dental readiness as predictors

					Correlations		
Predictor variable*	B^†^	Standard error	β^‡^	*P*	zero-order	partial	semi partial
Constant	2.785	1.372		0.043			
Years in military service	0.224	0.052	0.203	**<0.001**	0.192	0.212	0.201
Dental readiness (0 = unready, 1 = ready)	-1.409	0.678	-0.099	**0.038**	-0.129	-0.105	-0.097
Dental anxiety	0.470	0.125	0.179	**<0.001**	0.200	0.187	0.176
Somatization	0.241	0.201	0.114	0.232	0.206	0.061	0.056
Obsessive-compulsive	0.470	0.269	0.180	0.082	0.212	0.088	0.082
Interpersonal sensitivity	0.258	0.342	0.072	0.450	0.197	0.038	0.035
Depression	-0.082	0.269	-0.031	0.761	0.172	-0.015	-0.014
Anxiety	-0.036	0.363	-0.013	0.922	0.174	-0.005	-0.005
Hostility	0.112	0.262	0.038	0.669	0.178	0.022	0.020
Phobia	-0.259	0.388	-0.069	0.504	0.156	-0.034	-0.031
Paranoid ideas	0.258	0.217	0.096	0.236	0.178	0.060	0.056
Psychoticism	-0.555	0.338	-0.165	0.102	0.134	-0.083	-0.077

*R = 0.383, R2 = 0.147, Adjusted R2 = 0.120, F = 5.576, *P* < 0.001. Significant values are in bold.

†Unstandardized coefficient.

‡Standardized coefficient.

Global indices of psychopathology, GSI and PST, were significant predictors of OHRQoL, while PSDI was not (Table 7). However, the indices accounted for less than 5% of variability. The addition of indices in the regression models significantly weakened the predictive value of dental readiness. Participants who did not report a single symptom from Positive Symptom Distress Index were not taken into PSDI analysis. Since GSI, PST, and PSDI showed multicollinearity problem, a separate model was created for each of them.

**Table 7:** Multiple regression analysis models for prediction of oral health-related quality of life based on Oral Health Impact Profile sum score which used global psychopathology indices, years spent in military service and dental readiness as predictors

Predictor variable	B*	Standard. error	β†	*P*	Correlation		
					zero-order	partial	semipartial
Constant 1^‡^	3.260	1.343		0.016			
Dental readiness (0 = unready, 1 = ready)	-1.290	0.676	-0.091	0.057	-0.129	-0.095	-0.090
Years in military service	0.219	0.052	0.199	<0.001	0.192	0.208	0.199
Global Severity Index	3.323	0.823	0.190	<0.001	0.202	0.199	0.190
Dental anxiety	0.471	0.125	0.179	<0.001	0.200	0.186	0.177
Constant 2^§^	3.117	1.333		0.020			
Dental readiness (0 = unready, 1 = ready)	-1.252	0.671	-0.088	0.063	-0.129	-0.093	-0.087
Years in military service	0.220	0.051	0.200	<0.001	0.192	0.210	0.199
Positive Symptoms Total	0.119	0.025	0.223	<0.001	0.243	0.232	0.221
Dental anxiety	0.439	0.125	0.167	<0.001	0.200	0.174	0.164
Constant 3^║^	2.908	2.139		0.175			
Dental readiness (0 = unready, 1 = ready)	-1.150	0.780	-0.079	0.141	-0.115	-0.082	-0.078
Years in military service	0.215	0.061	0.189	<0.001	0.172	0.192	0.188
Positive Symptom Distress Level	1.030	1.037	0.053	0.321	0.018	0.055	0.053
Dental anxiety	0.523	0.140	0.203	<0.001	0.194	0.203	0.199

*Unstandardized coefficient.

†Standardized coefficient.

‡R = 0.228; R2 = 0.052; Adjusted R2 = 0.047; F = 10.935; *P* < 0.001.

§R = 0.352; R2 = 0.124; Adjusted R2 = 0.115; F = 14.069; *P* < 0.001.

^║^R = 0.286; R2 = 0.082; Adjusted R2 = 0.071; F = 7.7235; *P* < 0.001.

## Discussion

This research indicated that OHRQoL instrument had the ability to distinguish classes of dental readiness. Class 3 soldiers had higher frequency of psychological discomfort, psychological disability, and physical pain and handicap than Class 1 soldiers. Although those OHRQoL dimensions accounted for great proportion of variability, they correctly classified less than 40% of participants. These findings indicate that, although there was a trend of deterioration of OHRQoL with lower dental readiness, OHRQoL is a multidimensional category that is moderately associated with the clinically assessed dental readiness. This is corroborated by the fact that in multiple linear regression, when all other variables in the model were controlled for, dental readiness was a significant but weak predictor of OHRQoL as it accounted for only 1% of OHRQoL variability. Thus, OHRQoL instruments could be used for detecting those Class 2 soldiers who due to the impaired QoL should have priority in the sanation of dental conditions. This could reduce the chances of their reporting decreased combat readiness because of QoL impairment, increase the unit’s efficiency, and reduce the costs and risks of transport to medical treatment.

Dental unreadiness was reported to be a major problem for US troops, where up to 45% of soldiers needed dental work before deployment (23). Before the Croatian accession to NATO, more than 60% of Croatian soldiers were classified as Class 3 (5) and in this study, conducted after the accession to NATO, this share was reduced to about 1/3 of soldiers. A probable reason for this improvement was the soldiers' motivation to improve their oral health in order to be permitted to participate in international peacekeeping missions. OHRQoL indicators are subjective measures that provide information on the impact of oral conditions on everyday activities and they include damage, discomfort, functional limitations, disability, and handicap. Therefore, OHRQoL assessment should be an integral part of oral health assessments and should be measured before the deployment of soldiers. This study indicated that age, military experience, education, dental readiness, and psychological dimensions had a small role in the prediction of OHRQoL and accounted for a small part of variability. Demographic and military variables and dental readiness together accounted for 5.4% of variability, and psychological dimensions, military experience, and dental readiness accounted for 12%. The weak association between the clinical status of oral health and OHRQoL was also found in a systematic review (24). It was also found that QoL was significantly affected by the number of functional teeth (healthy and filled) (25). Other clinical measures of oral health were associated to specific dimensions of QoL but not with the general effect. Components of Decayed Missing Filled Teeth index, average periodontal attachment loss, loss of frontal teeth, and the number of teeth in functional occlusion were weak but significant predictors of OHRQoL (26). The loss of frontal teeth had a greater influence on OHRQoL, regardless of whether prosthodontic restoration was performed, while the loss of lateral teeth had an influence only if dental arch was interrupted and was not prosthodontically restored. A complete lack of teeth had an influence on OHRQoL in the OHIP subscales of functional limitation, physical and social disability, and handicap (27). Although the number of extracted teeth was the strongest clinical predictor of OHRQoL, it only accounted for 18% of OHIP variability, while general health, stress, financial income, age, and type of health insurance accounted for additional 14% (28). Other OHRQoL instruments found similar results (29).

Different levels of oral health had different effects on the QoL, since clinical indicators are often mediated and modified by functional and experiential factors such as mastication ability and pain, and by sociodemographic, cultural, economic, and psychological factors (26,30). The present study indicated that, although psychological dimensions correlated significantly with OHRQoL, with worse QoL being associated with greater intensity of psychological symptoms, the correlation was moderate or weak (r <0.5). Dental anxiety, somatization, obsessive-compulsive symptoms, and interpersonal sensitivity had a somewhat stronger correlation (r >0.20) than other psychological dimensions. Although obsessive-compulsive symptoms showed the strongest individual correlation with OHRQoL, they did not have a significant predictive role in the multivariate model. Only dental anxiety was a significant predictor in explaining the variability of OHRQoL, yet it accounted for only 3% of the variability. Increased dental anxiety and dental unreadiness were related to worse OHRQoL. OHRQoL instruments adapted to specific orodental conditions have been suggested to be more useful in the assessment of OHRQoL than generic instruments designed for the purpose (30).

All dimensions of OHIP have been suggested to be significantly impaired by temporomandibular dysfunctions; however, even greater impairment was reported in patients with dental anxiety (31-33). This is in line with our findings of dental anxiety as a significant predictor of OHRQoL.

Although we expected that somatization and depression would best predict poor OHRQoL, in the univarate model OHIP showed the highest correlation with obsessive-compulsive symptoms. Obsessive-compulsive persons, due to their tendency toward perfectionism, probably consider smaller oral health problems as considerable QoL impairment.

The face, mouth, and lower jaw are often the manifestation sites of psychological conditions, as is the case in burning mouth syndrome and temporomandibular dysfunction (34). Since depression and somatization are generally recognized as important variables in diagnosing and treating orofacial pain, we expected that they would be an important predictor of OHRQoL (35,36). However, this was not the case. Despite the finding that single psychological dimensions were not significant predictors of OHRQoL, the overall psychological distress level and the number of self-reported symptoms were. To a certain extent we expected this because perceived global symptomatology, rather than specific psychological symptoms, is more likely to be associated with the summary measure of OHRQoL.

The identified risk factors may be important predisposing, cumulative, repetitive, and prognostic factors that should be taken into consideration in military public health measures. Theoretical models have provided a framework for examining contributive and potentially confounding factors in the relation between going to war mission and the HRQoL (10,37).

In spite of the introduced dental readiness classification, it appears that even up to 70% of dental problems in military personnel are nonpreventable, ie, they had not been not indicated for urgent treatment at the previous annual dental examination (38). Even if all urgent dental procedures are previously finished, up to 92 dental emergencies per 1000 soldiers per year can be expected (38).

Although this study found a moderate association between OHRQoL and clinical, military, demographic, and psychological variables, the significant predictors could be used as a basis for further research of clinical and psychosocial factors of OHRQoL.
